# Utilization of biotechnological drugs in rare diseases requiring the use of off-label drugs in children in Turkey

**DOI:** 10.3906/sag-2012-355

**Published:** 2021-08-30

**Authors:** N. İpek KIRMIZI, Volkan AYDIN, Narin AKICI, Banu BAYAR, Ahmet AKICI

**Affiliations:** 1 Department of Medical Pharmacology, School of Medicine, Istanbul Medipol University, İstanbul Turkey; 2 Department of Medical Pharmacology, International School of Medicine, Istanbul Medipol University, İstanbul Turkey; 3 Department of Pediatrics, Haydarpasa Numune Training and Research Hospital, İstanbul Turkey; 4 Turkish Medicines and Medical Devices Agency, Ministry of Health, Ankara Turkey; 5 Department of Medical Pharmacology, School of Medicine, Marmara University, İstanbul Turkey

**Keywords:** Off-label drug use, pediatrics, rare disease, biotechnological drugs, canakinumab

## Abstract

**Background/aim:**

Pediatric patients, especially those with rare diseases, represent a population that has a high tendency towards off-label drug use (OLDU) and needs a more careful practice of pharmacotherapy than in adults. We aimed to investigate biotechnological drug use in children with rare diseases requiring OLDU.

**Materials and methods:**

This retrospective study examined all single-diagnosed OLDU applications (n = 5792) for 4992 children (<18-year) in Turkey. Applications of rare diseases were selected, and their descriptive characteristics were examined, including demographic features of patients, biotechnological drug utilization status, and disease categories. The off-label statuses of the drugs at the end of 2020 were also examined.

**Results:**

In total, 77.7% (n = 4501) of OLDU applications were made for rare diseases. Biotechnological drug use was higher in rare disease applications than in nonrare diseases (37.9% vs. 19.2%, respectively; p < 0.0001). Canakinumab was the top applied biotechnological drug (73.2%). Compared to that in small-molecule drugs, the mean age of patients was higher in biotechnological drug-containing applications (8.1 ± 5.3 vs. 9.7 ± 4.9, respectively; p < 0.0001). Biotechnological drug use was higher in nonneoplastic rare diseases (40.3%) than in neoplastic rare diseases (26.4%), (p < 0.0001). At the end of 2020, the approval status of the off-label indications covered in 2016 was significantly higher for rare (24.4%) vs. nonrare (5.2%, p < 0.0001) diseases and for biotechnological (32.3%) vs. small-molecule (13.9%, p < 0.0001) drugs. In total, 87.7% of the drugs would have to be still used in the off-label setting at the end of 2020.

**Conclusion:**

It was seen that more than three-quarters of the pediatric OLDU applications are for rare diseases, and the need for biotechnological OLDU in this group is almost 2-fold of small-molecule drug use. While further projected findings imply a higher approval tendency for rare diseases and biotechnological drugs, there seems to be more room for improvement for pediatric drug use.

## 1. Introduction

Despite differences in its definition, rare diseases are generally considered to cover diseases affecting no more than 5/10.000 people in the community [1]. Studies show that rare diseases in childhood may cause sequelae in children and pose a serious psychological and economic burden on both families and all stakeholders of the health system [1,2]. A major attempt to alleviate this burden is obviously the novel drug development studies for treating rare diseases. However, apart from the conductibility of a limited number of studies in children due to ethical reasons, it is known that clinical studies in rare diseases have additional methodological challenges and the pharmaceutical industry does not always find it attractive to direct research and development incentives to this group [3,4]. Although granting extra privileges to clinical trials for rare diseases has been on the agenda of health authorities for a long time, off-label drug use (OLDU) remains prevalent in diagnoses within the scope of rare diseases [3–5]. Moreover, it is estimated that the high OLDU burden already existing in children may escalate even more in the case of rare diseases [3,6–8].

Data on pediatric usage of biotechnological drugs is very limited [9]. A biotechnological drug is defined as “a human medicinal product whose active ingredients or substances were produced in or purified from a biological source, where the quality, manufacturing process, and audits are demonstrated through physicochemical and biological tests” [10]. Biotechnological drugs are among the treatment options in a wide range of conditions, including cancers, rheumatological/immunological diseases, and nephrological diseases, and they can also be used in the treatment of many rare diseases in these areas [11,12]. Available literature has not revealed any study about the management of rare diseases via off-label drugs with a particular focus on biotechnological products [1,3]. In fact, biotechnological drug utilization data is needed in rare diseases to overcome therapeutic challenges in these diseases, especially in the pediatric population. In this study, we aimed to evaluate the use of biotechnological drugs for rare diseases in children who needed off-label drugs.

## 2. Materials and Methods

### 2.1. Data collection

In this cross-sectional study, we examined OLDU applications conducted between 1 January and 31 December 2016 in Turkey. Applications for OLDU are submitted to the Turkish Medicines and Medical Devices Agency (TMMDA) of the Ministry of Health to be evaluated and approved [13]. We reviewed single-diagnosed applications for the pediatric population (<18 years) submitted by pediatricians, subspecialists of pediatrics, child psychiatry, and pediatric surgeons in 2016 (n = 8272). In this dataset, those applications belonging to a patient for the same drug during the study period were determined as a repeated application and excluded (n = 2480).

The applications were grouped according to their indicated diagnoses as rare and nonrare diseases. Whether the diseases in the applications were rare or not were decided according to the Orphanet database supported by the European Commission [14]. The biotechnological or small-molecule statuses of the applied drugs were determined, and their distributions were compared according to the groups. In addition, single or multiple drug application statuses were compared in OLDU applications for rare or nonrare diseases. The age and sex of the patients and the distribution of the physician’s specialties were compared according to the diagnosis groups. 

OLDU applications for rare diseases were further analyzed in detail. The drugs in these applications were grouped according to the “Anatomical Therapeutic Chemical” (ATC) coding system. The first five most common biotechnological and small-molecule active substances were identified. The distribution of the drugs at ATC-1 level was evaluated, and the percentage of biotechnological or small-molecule drugs in each sublevel was determined. In addition, ATC-1 level status within biotechnological and small-molecule drugs was evaluated. The most frequently applied biotechnological and small-molecule drug in an off-label setting was determined for each ATC-1 group. The mean ages of patients for whom OLDU was applied and their biotechnological and small-molecule drug statuses were compared for each ATC-1 group.

The distributions of the main diagnosis groups according to “International Statistical Classification of Diseases (ICD)” in rare disease applications were examined according to their status as biotechnological or small-molecule drugs. The most frequently applied biotechnological or small-molecule drugs were examined in each main diagnosis group. The diagnoses were also classified as neoplastic and nonneoplastic and were compared in terms of off-label biotechnological drug use. Single/multiple OLDU applications were compared to determine whether these were made for biotechnological drugs or not. These applications were also evaluated in terms of off-label drugs’ pharmaceutical form and route of administration.

As the regulatory approval statuses of the drugs may alter by time due to the completion of trials, we also examined the off-label statuses of the drugs at the end of 2020 to reflect the progression of the safe and effective use of drugs for children. For this purpose, the approval statuses of all drugs applied for all off-label indications in 2016 were analyzed as if they were applied at the end of 2020. This projection was detailed for rare vs. nonrare diseases and for biotechnological vs. small-molecule drugs.

### 2.2. Statistical analysis

Statistical analyses were made through SPSS 24.0 software. Categorical variables were expressed as number/percentage, and continuous variables were mentioned as mean/standard deviation. The comparisons between categorical and continuous variables were analyzed via chi-square and t-test, respectively. An overall 5% type-I error level was used to infer statistical significance.

### 2.3. Ethical approval

The data were collected after the study was approved by Ethics Committee of Institute of Health Sciences of Marmara University (Approval No: 11.09.2017/171).

## 3. Results

### 3.1. Main findings

A total of 5792 OLDU applications were detected for 4992 children in the study period. It was determined that the applications with rare diseases constituted 77.7% (n = 4501) and that these applications were made for 3894 (78.1%) patients. There were no significant differences between children with rare diseases and nonrare diseases in their applications (p > 0.05) in terms of age (8.6 ± 5.1 and 8.5 ± 5.0, respectively) and sex (male, 52.4% and 55.4%; respectively). The need for multiple distinct off-label drugs for one patient during the year was similar for rare and non-rare diseases (p > 0.05). The percentage of biotechnological drug use for rare diseases was significantly higher than that for nonrare diseases (37.9% and 19.2%, respectively), (p < 0.0001), (Table 1). OLDU applications were made most frequently by pediatric rheumatologists/nephrologists (28.1%) and child neurologists (39.0%) in rare and nonrare diseases, respectively.

**Table 1:** Patient and drug characteristics of OLDU applications based on the rare disease status of the diagnoses.

		Indication for off-label drug use	
		Rare disease	Nonrare disease
Mean age, years (±SD)*		8.6 ± 5.1	8.5 ± 5.0
Sex*	Male	2041 (52.4)	608 (55.4)
	Female	1853 (47.6)	490 (44.6)
OLDU applications for	Single drug	3412 (75.8)	961 (74.4)
	Multiple drugs	1089 (24.2)	330 (25.6)
Class of drug§	Small-molecule	2797 (62.1)	1043 (80.8)
	Biotechnological	1704 (37.9)	248 (19.2)
Total OLDU applications, n (%)		4501 (77.7)	1291 (22.3)

OLDU, off-label drug use. *Age and sex data were based on the number of patients (n = 4992); §p < 0.0001.

### 3.2. Rare diseases

**Figure 1:**
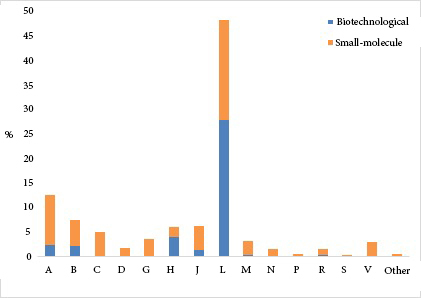


When the drugs in the applications were examined at the ATC-1 level, the most commonly encountered groups were “L-Antineoplastic and immunomodulating agents” (48.2%), “A-Alimentary tract and metabolism” (12.4%), and “B-Blood and blood-forming organs” (7.4%). When biotechnological drug ratio was examined in each subgroup at ATC-1 level, the highest rate was “H-Systemic hormonal preparations” (63.2%), followed by “L-Antineoplastic and immunomodulating agents” (57.5%) and “B-Blood and blood-forming organs” (27.5%) (Figure 1).

When the distribution of biotechnological and small-molecule drugs in the applications was analyzed at the ATC-1 level, it was found that both biotechnological and small-molecule drugs more commonly belong to the “L-Antineoplastic and immunomodulating agents” group (73.2% and 33.0%, respectively). Canakinumab and mycophenolate were the most common biotechnological (20.8%) and small-molecule (29.6%) drugs in this group. The mean age of the patients using biotechnological drugs (9.7 ± 4.9 years) was significantly higher than that of the patients using small-molecule drugs ((8.1 ± 5.3 years; p < 0.0001). This age difference between biotechnological and small-molecule drugs was preserved in patients using “A-Alimentary tract and metabolism” (p < 0.0001), “J-Antiinfectives for systemic use” (p = 0.008), and “M-Musculoskeletal system” drugs at ATC-1 level (p = 0.025), (Table 2).

**Table 2:** Distribution of ATC-1 groups based on the biotechnological and small-molecule status of drugs in rare disease applications.

ATC-1 code	Biotechnological drugs			Small-molecule drugs		
	n, (%)	Mean age ± SD	The most frequent drug, (%)	n, (%)	Mean age ± SD	The most frequent drug, (%)
A	103, (6.0)	9.1 ± 3.7#	Elosulfase alfa, (89.3)	454, (16.2)	5.0±4.5	Sapropterin, (42.5)
B	92, (5.4)	8.8 ± 5.2	Coagulation factor VIIa, (32.6)	242, (8.7)	9.2±4.9	Iloprost, (59.1)
C	-	-	-	219, (7.8)	8.2±5.3	Bosentan, (33.8)
D	-	-	-	73, (2.6)	4.5±3.3	Isotretinoin, (95.9)
G	-	-	-	161, (5.8)	8.6±5.4	Sildenafil, (54.0)
H	172, (10.1)	9.3 ± 4.3	Somatropin, (99.4)	100, (3.6)	9.2±5.2	Hydrocortisone, (28.0)
J	60, (3.5)	8.6 ± 5.7#	Intravenous immunoglobulin, (86.7)	216, (7.7)	6.4±5.2	Valganciclovir, (37.5)
L	1248, (73.2)	9.8 ± 5.0	Canakinumab, (20.8)	923, (33.0)	10.0±4.9	Mycophenolate, (29.6)
M	12, (0.7)	12.6 ± 5.0#	Denosumab, (100,0)	128, (4.6)	8.8±3.8	Ataluren, (35.2)
N	-	-	-	68, (2.4)	9.2±4.9	Trihexyphenidyl, (22.1)
P	-	-	-	16, (0.6)	1.9±3.6	Pyrimethamine, (100.0)
R	13, (0.8)	9.3 ± 5.9	Omalizumab, (53.8)	52, (1.9)	7.9±5.1	Mannitol, (51.9)
S	-	-	-	3, (0.1)	11.3±9.1	Dexamethasone, (66.7)
V	-	-	-	126, (4.5)	6.6±5.5	Calcium folinate, (52.4)
Others*	4, (0.2)	10.5 ± 4.8	-	16, (0.6)	7.5±6.0	-
Total	1704, (100.0)	9.7 ± 4.9#	Canakinumab, (15.3)	2797, (100.0)	8.1±5.3	Mycophenolate, (9.8)

A, Alimentary tract and metabolism; B, Blood and blood-forming organs; C, Cardiovascular system; D, Dermatological; G, Genito urinary system and sex hormones; H, Systemic hormonal preparations, excl. sex hormones and insulins; J, Antiinfectives for systemic use; L, Antineoplastic and immunomodulating agents; M, Musculoskeletal system; N, Nervous system; P, Antiparasitic products, insecticides, and repellents; R, Respiratory system; S, Sensory organs; V, Various; *, Applications that have no defined ATC code. # p < 0.05 biotechnological vs. conventional drugs group.

The most common five biotechnological drugs used off-label were canakinumab (15.3%), rituximab (13.3%), eculizumab (11.2%), somatropin (10.0%), and anakinra (7.9%). In small-molecule drugs, this ranking included mycophenolate (9.8%), sapropterin (6.9%), iloprost (5.1%), sirolimus (4.0%), and tacrolimus (3.5%). “Endocrine, nutritional and metabolic diseases” main ICD group were the most common indications for both biotechnological (33.9%) and small-molecule drugs (27.2%). The most commonly encountered biotechnological and small-molecule drug in this diagnosis group was canakinumab (31.7%) and sapropterin (25.1%), respectively (Table 3).

**Table 3:** Distribution of main diagnosis groups based on their biotechnological or small-molecule drug status.

Main diagnosis group	Biotechnological drugs		Small-molecule drugs	
	n (%)	The most frequent drug, (%*)	n (%)	The most frequent drug, (%*)
Certain infectious and parasitic diseases	12 (0.7)	Interferon beta-1a, (41.7)	85 (3.0)	Valganciclovir, (44.7)
Neoplasms	208 (12.2)	Bevacizumab, (21.6)	579 (20.7)	Isotretinoin, (11.9)
Diseases of the blood and blood-forming organs and certain disorders involving the immune mechanism	350 (20.5)	Eculizumab, (50.0)	222 (7.9)	Eltrombopag, (26.6)
Endocrine, nutritional and metabolic diseases	578 (33.9)	Canakinumab, (31.7)	761 (27.2)	Sapropterin, (25.1)
Diseases of the nervous system	34 (2.0)	Rituximab, (41.2)	205 (7.3)	Ataluren, (22.0)
Diseases of the eye and adnexa	2 (0.1)	Rituximab, (100.0)	14 (0.5)	Idebenone, (35.7)
Diseases of the circulatory system	6 (0.4)	Dornase alfa, (50,0)	431 (15.4)	Iloprost, (32.7)
Diseases of the respiratory system	5 (0.3)	Dornase alfa, (40,0)	2 (0.1)	Pamidronic acid, (50.0)
Diseases of the digestive system	14 (0.8)	Denosumab, (50.0)	22 (0.8)	Mycophenolate, (36.4)
Diseases of the skin and subcutaneous tissue	5 (0.3)	Intravenous immunoglobulin, (20.0)	9 (0.3)	Flutamide, (44.4)
Diseases of the musculoskeletal system and connective tissue	330 (19.4)	Adalimumab, (29.7)	89 (3.2)	Mycophenolate, (69.7)
Diseases of the genitourinary system	127 (7.5)	Rituximab, (84.3)	249 (8.9)	Mycophenolate, (56.6)
Certain conditions originating in the perinatal period	1 (0.1)	Factor VIII inhibitor bypassing activity, (100.0)	54 (1.9)	Calcium folinate, (29.6)
Congenital malformations, deformations, and chromosomal abnormalities	32 (1.9)	Somatropin, (96.9)	75 (2.7)	Sirolimus, (36.0)
Total	1704 (100.0)	Canakinumab, (15.3)	2797 (100.0)	Mycophenolate, (9.8)

*Percentage of drugs that are used in the diagnosis group column below.

**Figure 2:**
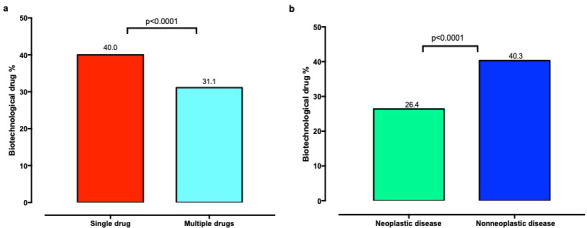


It was detected that 17.5% of the rare diseases were neoplastic in nature. The use of biotechnological drugs was significantly lower in neoplastic diseases (26.4%) than that in nonneoplastic ones (40.3%), (p < 0.0001). In rare diseases requiring multiple OLDU applications, biotechnological drug utilization (31.1%) was significantly lower compared to that in the diseases which needed only a single off-label drug (40.0%), (p < 0.0001), (Figures 2a and 2b).

Analysis of off-label drug administration routes showed that the drugs were administered with injectable forms (49.1%), followed by enteral (47.1%) and other administration routes (3.8%). In almost all biotechnological drug applications, the route of administration was injection (99.6%), whereas the injectable forms constitute 18.2% of small-molecule drugs.

### 3.3. Projected off-label status

We identified that 20.1% (n = 1163) of the off-label indications covered in 2016 would have been approved as of 1January 2021. This projection was significantly more pronounced for rare diseases (24.4%) than nonrare diseases (5.2%, p < 0.0001) and for biotechnological drugs (32.3%) than small-molecule drugs (13.9%, p < 0.0001). In terms of all drugs applied for off-label use in 2016 (n = 353), twelve drugs (3.4%) had been approved for those particular indications until 2021, whereas 33 drugs (9.3%) were partially approved for certain indications. The rest (87.3%) was observed to be used in the off-label setting until the end of 2020.

## 4. Discussion

The present study was the first to focus on off-label biotechnological drug use in children at the national level, where important findings that may help to manage rare diseases were obtained. The pediatric population requires additional care in drug use and frequently needs OLDU [3]. The management of rare diseases in this group, which already has limited drug use data, is a special area with unique balances. Despite the incentives for clinical trials in rare diseases, the number of drugs approved for use in these diseases remains low [15,16]. Considering that 95% of rare diseases do not have approved treatment, it is often expected to refer to OLDU in rare diseases in children [15,17]. This was supported by our finding that more than three-quarters of OLDU applications in children were rare diseases. Although rare diseases are thought to affect a restricted population, 8% of the society was reported be faced with a congenital or acquired rare disease [2,18]. Around 8000 rare diseases have been described worldwide, 70% of which had a pediatric onset [15,17]. Considering the population of children in Turkey [19], rare diseases (n = 3894) that need to be referred to the health authority to be managed by off-label drugs can affect 17 out of every 100,000 children. 

Similar to this study, another study conducted in Turkey in 2015 on the use of OLDU in children reported that applications were made more frequently for male patients [8]. Although this may suggest a country-specific difference at first glance, studies in other countries based on drug use in children also reported similar findings, especially in younger children [20,21]. 

Biotechnological drug use is on an increasing trend across the globe. Similarly, studies focusing on new drug development show that the number of biotechnological drugs increases with a rising momentum [22,23]. It is known that many new drugs have been developed in recent years, and biotechnological methods have been used in this process, especially in rare diseases, which are a common area of need for alternative treatment [15,23]. In our study, the use of off-label biotechnological drugs in rare diseases is approximately twice that of nonrare diseases, indicating the need for biotechnological drugs to manage rare diseases in children. Although there is not enough data on the overall biotechnological drug utilization in children, it has been reported that they are frequently used in immunocompromised conditions and childhood cancers are one of the areas where biotechnological drug use is most needed [9]. Considering the area covered by these conditions in rare diseases, it can be said that the findings obtained in our study are partially compatible with the literature. On the other hand, it can be stated that when off-label drugs are required in rare diseases, it is important that more than one of the three drugs applied is biotechnological in terms of quantitative representation of the need in this area. This high utilization rate and further pharmacoepidemiological study findings are expected to provide the basis for the on-label use of biotechnological drugs in rare diseases. Moreover, despite this increasing trend of biotechnological drug use in rare diseases, information on the pharmacokinetics, pharmacodynamics, and pharmaceutical application of these drugs is still limited in children, especially in those with rare diseases, compared to small-molecule drugs [9]. Besides, the use of biotechnological drugs other than the indications for which they are licensed takes place through extrapolation [24]. Considering their use in rare diseases, it may be suggested that the existing risk management plans of these drugs may be more likely to accompany various uncertainties in rare disease-oriented practice and that additional challenges may arise, indicating the need for additional measures.

Consistent with the results of the previously reported OLDU-focused study in Turkey, we determined that nearly half of the drugs applied for rare diseases belonged to the antineoplastic and immunomodulating agents [7]. This main group was ranked first in both biotechnological and small-molecule drugs for rare disease applications. This may have been due to the procedure related to the application of OLDU in Turkey, rather than the fact that biotechnological drug use in rare diseases brought this main group to the top rank. In fact, the literature findings outside Turkey showed that the most commonly encountered off-label used drugs in children indicated other drug groups [6,25]. This can be attributed to the diversity of different countries in OLDU-related practices. For instance, drugs in antineoplastic and immunomodulating agents group can be used after meeting more rigorous clinical requirements than other drugs due to their difficulty in safety, cost, and compliance [9]. The fact that the OLDUs included in our study did not cover “routine use of off-label drugs that do not require additional procedures” but consisted of OLDUs that are subject to the application to the health authority may have led to this risky drug group being the main group most commonly encountered.

Although the definition of orphan drugs covers drugs from different groups for different reasons, it is a commonly used concept for many drugs used to treat rare diseases due to its small market share [16]. It is noteworthy that in our study, the most common biotechnological drug was canakinumab and the most common small-molecule drug was mycophenolate. In addition, canakinumab was observed to be the most common biotechnological drug in applications containing endocrine, nutritional and metabolic diseases, the most common diagnostic group. In fact, both these drugs were reported to be at the top in other studies on OLDU in Turkey [7,8,26]. Canakinumab, a recombinant human monoclonal antibody, is an interleukin-1 beta inhibiting orphan drug. Systemic juvenile idiopathic arthritis and extremely rare cryopyrin-associated periodic syndromes (CAPS) constitute the main indications of this drug [27]. Another monoclonal antibody, eculizumab, which was reported to be the most frequently encountered drug in pediatric OLDU practice in Turkey [8], ranked third in our study.

The mean age of the patients requiring off-label biotechnological drugs was higher than that of those requiring small-molecule drugs. This might be attributed to several reasons. The first may be that the diagnosis of diseases requiring biotechnological drugs, such as amyloidosis, which is common in OLDU applications, can be diagnosed in older age groups [8,9,28]. Another important reason is that access, application, awareness, cost-effectiveness, reimbursement condition, etc. aspects of the small-molecule drugs can bring up their use before biotechnological drugs [1,29]. Therefore, this age-related condition can be explained by the fact that physicians are likely to apply for biotechnological drugs later for children with rare diseases requiring OLDU.

The status of neoplastic cases in rare diseases is another debatable point of the research. It was reported that 11.1% of rare diseases identified in Orphanet were neoplastic [18]. We found that around one-sixth (17.5%) of rare diseases have neoplastic origin. We further observed that biotechnological drugs were required less frequently for rare neoplastic diseases than nonneoplastic ones. Various studies reported that neoplastic diseases constituted the main indication group with the highest number of drug development studies and orphan drug approval among rare diseases [4,16,23]. This difference seems to suggest that there may be more alternative drugs for the treatment of rare neoplastic diseases.

The high share of nearly 80% covered by rare diseases in OLDU raises the question of whether the management of rare diseases with OLDU is routine or a mandatory practice arising from seeking treatment. While the method of the study did not allow for a direct answer to this, we observed that one off-label drug was sufficient for at least 1 year in three out of every four applications in both rare and nonrare diagnoses. This shows the tendency of the situation in the diseases that need OLDU to be partially controlled with a single off-label drug. Accordingly, it can be inferred that management of these diseases could be maintained routinely with OLDU, rather than seeking treatment, at least during the study period, but this was not unique to rare diseases. On the other hand, it was determined that biotechnological drug use in rare diseases requiring multiple applications of OLDU was lower than those requiring application for a single drug. This difference may be related to the tendency of physicians not to replace biotechnological drugs or not to add new biotechnological drugs in the same year in a sensitive therapeutic area like rare diseases. In other words, it can be said that although biotechnological drugs are often used in rare diseases, physicians tend to insist more on their off-label drug choices. These findings highlight the importance of investigating the use of off-label biotechnological drugs in rare diseases.

The projected approval status of the OLDU showed that one-fifth of this special drug use turned into routine clinical practice between 2016 and 2021. We observed that this possibility was near 5-fold higher in rare diseases and 2.3-fold higher for biotechnological drugs, which seems to be consistent with ongoing efforts in these therapeutic areas [15,16,23]. On the other hand, the fact that only 12.7% were approved or partially approved during 4 years may suggest that there is further room for improvement for pediatric OLDU.

Various research reported that the proportion of parenteral drugs prescribed by routine prescribing procedure in Turkey was between 4% and 6% [30]. However, in OLDU, which is a nonroutine application, this rate was reported to escalate up to 38% [7], with no data on the pediatric population. In our study, the distribution of injectable forms found in rare diseases in children increased approximately 10 times the routine practice, and the share of enteral and injectable pharmaceutical forms was almost equal. This situation can be partly explained by the fact that more than 1/3 of the drugs used to manage rare diseases with OLDU were biotechnological drugs in our study. We further noticed that nearly all biotechnological drugs were administered to the patient in injectable forms. In fact, although various studies are underway to deliver biotechnological drugs in different pharmaceutical forms, intravenous and subcutaneous routes are still the most common ways of administration in biotechnological drugs [31]. On the other hand, we observed that the proportion of injectable drugs in off-label use of small-molecule drugs in children was approximately five times higher than that of routine use. This suggests that injectable pharmaceutical forms are more common in OLDU settings than in routine practice, even considering only small-molecule drugs.

The main limitation of this research was that all of the data accessed were OLDU applications with no other medical records of patients. Such lack of data may underestimate the actual reasons that may have led the physician to apply for OLDU, including prior drug use experiences for this indication, other drug use-related ineffectiveness/adverse effects experience or comorbid conditions. In addition, the use of the Orphanet database in the classification of rare diseases can be considered a relative limitation because the rare diseases that do not exist in this database could not be included. 

In conclusion, the details of particularly biotechnological product-oriented OLDU in pediatric rare diseases were described for the first time at a country-level. It is observed that the majority of OLDU applications made for children consist of rare diseases and that physicians tend to prefer biotechnological drugs more often in these diseases. While projected findings imply a higher approval tendency towards rare diseases and biotechnological drugs, the need for improvement still seems to remain for routine safe and effective pediatric drug use. Important findings obtained in the study, especially those specific to biotechnological drugs, are expected to help manage the pharmacotherapy process in this fragile population in a way that would be less likely to require off-label drugs.

## Funding

This work has been supported by Marmara University Scientific Research Projects Coordination Unit under grant number SAG-C-DRP-150218-0034.

## Informed consent

The study design does not require obtaining informed consent.

## References

[ref1] (2010). Rare Diseases and Orphan Products: Accelerating Research and Development.

[ref2] (2015). Rare diseases are a “common” problem for clinicians. Australian Family Physician.

[ref3] (2007). Promoting Safety of Medicines for Children.

[ref4] (2017). State of Paediatric Medicines in the EU: 10 years of the EU Paediatric Regulation.

[ref5] (2010). Reforming off-label promotion to enhance orphan disease treatment. Science.

[ref6] (2018). Off-label and unlicensed drug use in children population. Therapie.

[ref7] (2017). A nationwide evaluation of off-label drug utilization in Turkey. Turkish Journal of Medical Sciences.

[ref8] (2020). Off-label drug use in pediatric patients: A comparative analysis with nationwide routine prescription data. Turk Journal of Pediatrics.

[ref9] (2012). Biologics in pediatrics.

[ref10] Türkiye İlaç ve Tıbbi Cihaz Kurumu. Biyobenzer Tıbbi Ürünler Hakkında Kılavuzu Taslağı.

[ref11] (2014). A better fit? Biotech versus Big Pharma in orphan/rare disease drug research. Expert Opinion on Orphan Drugs.

[ref12] (2018). Comparative analysis of legislative requirements about patients’ access to biotechnological drugs for rare diseases in central and Eastern European countries. Frontiers in Pharmacology.

[ref13] (2019). Endikasyon Dışı İlaç Kullanım Kılavuzu, 09 Şubat.

[ref14] (2017). Clinical Practice Guidelines for Rare Diseases: The Orphanet Database. PLoS ONE.

[ref15] (2013). Background Paper.

[ref16] Study on off-label use of medicinal products in the European Union 2017.

[ref17] (2019). The Lancet Diabetes & Endocrinology. Spotlight on rare diseases. Lancet Diabetes Endocrinology.

[ref18] (2020). Estimating cumulative point prevalence of rare diseases: analysis of the Orphanet database. European Journal of Human Genetics.

[ref19] (2016).

[ref20] (2008). Drug use in children: cohort study in three European countries. British Medical Journal.

[ref21] (1999). A one-year population-based study of drug prescriptions for Danish children. Acta Paediatrica.

[ref22] (2019). Evaluation of top-selling biotechnological medicine from 2003 to 2016 in Turkey. The Medical Bullin of Haseki.

[ref23] (2020). IMS Institute for Healthcare Informatics.

[ref24] (2018). The biosimilars journey: Current status and ongoing challenges. Drugs Context.

[ref25] (2018). Off-Label Medication Use in Pediatric and Neonatal Intensive Care Units: No Change Over a Decade. Advances in Therapy.

[ref26] (2018). Investigation of the Off-Label Drug Use at Provincial and Regional Levels. Gazi Medical Journal.

[ref27] Ilaris Prescribing information..

[ref28] (2018). Canakinumab treatment in children with familial Mediterranean fever: report from a single center. Rheumatology International.

[ref29] (2009). Controlling Off-label Medication Use. Annals of Internal Medicine.

[ref30] (2013). Akıcı A. Investigation of parenteral drug use in family health care centers across 32 provinces of Turkey. Anatolian Journal of Clinical Investigation.

[ref31] (2017). Disruption and maturity: The next phase of biologics.

